# Effect of risk status for severe COVID*-*19 on individual contact behaviour during the SARS-CoV-2 pandemic in 2020/2021—an analysis based on the German COVIMOD study

**DOI:** 10.1186/s12879-023-08175-2

**Published:** 2023-04-06

**Authors:** Jasmin Walde, Madhav Chaturvedi, Tom Berger, Antonia Bartz, Robin Killewald, Damilola Victoria Tomori, Nicole Rübsamen, Berit Lange, Stefan Scholz, Marina Treskova, Karolin Bucksch, Christopher I. Jarvis, Rafael Mikolajczyk, André Karch, Veronika K. Jaeger

**Affiliations:** 1 Institute of Epidemiology and Social Medicine, University of Münster, Albert-Schweitzer-Campus 1, 48149 Münster, Germany; 2 Department of Epidemiology, Helmholtz Centre for Infection Research, Braunschweig, Germany; 3 German Center for Infection Research, Braunschweig, Germany; 4 Immunization Unit, Infectious Disease Epidemiology, Robert Koch-Institute, Berlin, Germany; 5 Heidelberg Institute of Global Health, Heidelberg University Hospital, Heidelberg, Germany; 6 Institute for Medical Informatics, Statistics and Epidemiology (IMISE), University of Leipzig, Leipzig, Germany; 7 London School of Hygiene and Tropical Medicine, London, UK; 8 Institute for Medical Epidemiology, Biometrics and Informatics, Medical Faculty of the Martin Luther University Halle-Wittenberg, Halle, Germany

**Keywords:** Contact behaviour, SARS-CoV-2, Risk groups

## Abstract

**Background:**

One of the primary aims of contact restriction measures during the SARS-CoV-2 pandemic has been to protect people at increased risk of severe disease from the virus. Knowledge about the uptake of contact restriction measures in this group is critical for public health decision-making. We analysed data from the German contact survey COVIMOD to assess differences in contact patterns based on risk status, and compared this to pre-pandemic data to establish whether there was a differential response to contact reduction measures.

**Methods:**

We quantified differences in contact patterns according to risk status by fitting a generalised linear model accounting for within-participant clustering to contact data from 31 COVIMOD survey waves (April 2020-December 2021), and estimated the population-averaged ratio of mean contacts of persons with high risk for a severe COVID-19 outcome due to age or underlying health conditions, to those without. We then compared the results to pre-pandemic data from the contact surveys HaBIDS and POLYMOD.

**Results:**

Averaged across all analysed waves, COVIMOD participants reported a mean of 3.21 (95% confidence interval (95%CI) 3.14,3.28) daily contacts (truncated at 100), compared to 18.10 (95%CI 17.12,19.06) in POLYMOD and 28.27 (95%CI 26.49,30.15) in HaBIDS.

After adjusting for confounders, COVIMOD participants aged 65 or above had 0.83 times (95%CI 0.79,0.87) the number of contacts as younger age groups. In POLYMOD, this ratio was 0.36 (95%CI 0.30,0.43). There was no clear difference in contact patterns due to increased risk from underlying health conditions in either HaBIDS or COVIMOD. We also found that persons in COVIMOD at high risk due to old age increased their non-household contacts less than those not at such risk after strict restriction measures were lifted.

**Conclusions:**

Over the course of the SARS-CoV-2 pandemic, there was a general reduction in contact numbers in the German population and also a differential response to contact restriction measures based on risk status for severe COVID-19. This differential response needs to be taken into account for parametrisations of mathematical models in a pandemic setting.

**Supplementary Information:**

The online version contains supplementary material available at 10.1186/s12879-023-08175-2.

## Introduction

A key strategy in the response to the SARS-CoV-2 pandemic has been the population-wide reduction of contacts via various non-pharmaceutical interventions. In Germany, for instance, very strict contact reduction measures were put in place from March to May 2020. The summer of 2020 saw less intense restrictions, but stricter measures were reintroduced in November 2020 which were intensified in December 2020 in response to the second wave of the pandemic [1]. This was followed by a gradual easing of the restrictions starting from late spring 2021. Once diagnostic testing became widespread and vaccines were offered to the majority of the population, these restrictions were augmented to include individual vaccination or tested status. For example, from August 2021, most states in Germany introduced some variation on the so called ‘3G’ rules—individuals had to be either vaccinated, tested negative, or previously infected by SARS-CoV-2 and recently recovered to be able to access or use large parts of public life [2].

The effect of all these interventions on individual contact behaviour has been assessed in contact surveys, and population-wide reductions in the number of contacts have been reported for various countries [3–7]. However, these studies largely do not take into account differential risk of severe COVID-19 outcomes in different population subgroups.

One of the main goals of contact reduction measures is to protect people at increased risk of a severe course of COVID-19 to prevent morbidity and mortality, as well as the overloading of the healthcare system. Research indicates that people who perceived themselves to be at higher risk of severe COVID-19 made more efforts to reduce close contacts during the pandemic [8]. Further examination of differences in contact behaviour among different risk groups will provide valuable information for public health policy and communication, as well as the parametrisation of modelling studies. In this study, we provide a comprehensive analysis of contact behaviour during the pandemic, focusing on differences due to risk of severe COVID-19. We use data from the longitudinal contact survey COVIMOD [7], conducted in Germany over the first two years of the pandemic. We distinguish between groups with and without an increased risk of severe COVID-19, due to either old age or an underlying health condition. We also compare these data to data from pre-pandemic surveys to analyse if observed differences in contact patterns during the pandemic mirror underlying pre-pandemic differences or if they represent a differential response to contact reduction measures.

## Methods

### Contact surveys

#### COVIMOD

The contact survey COVIMOD (COVID Pandemic: Social Contacts and MODelling) was initiated in April 2020 in Germany. Participants were recruited via the market research company Ipsos from members of the online panel i-say.com [9], based on quotas about age, sex, and region to ensure that the study population broadly matches the German population with regards to distribution of sociodemographic characteristics. Some more information on how Ipsos creates samples can be found on their website [10]. A subgroup of adult participants with children under the age of 18 were invited to complete the survey as a proxy for their children. This allowed for the collecting of information about children’s social contacts.

The questionnaire for COVIMOD was based on the questionnaire of the CoMix study [4, 6] being conducted across various European countries. It includes questions on demographics, current behaviours, attitudes towards SARS-CoV-2, and the social contacts of participants. A version of the questionnaire, translated from German, can be found in Additional File 1.

A contact in COVIMOD is defined as “people who you met in person and with whom you exchanged at least a few words, or with whom you had physical contact”, which is in line with the pre-pandemic contact survey POLYMOD [11]. Participants were asked to list each contact individually for the first two survey waves of COVIMOD. From wave 3 onwards, in addition to individual contacts, participants could give aggregate group contacts in each of three different settings (work, school, and other), and for three contact age groups in each setting (< 18 years, 18 to 64 years, 65 years or older). All COVIMOD participants were also asked whether they had used a mask or public transport the previous day. In addition, participants were asked about their vaccination status from survey wave 17 onwards.

The timing and the sample sizes of the 31 survey waves included in this study can be found in Additional File 2, sheet 1.

#### POLYMOD and HaBIDS

We used the pre-pandemic POLYMOD (Improving Public Health POLicY in Europe through MODelling and Economic Evaluation of Interventions for the Control of Infectious Diseases) and HaBIDS (Hygiene and Behaviour Infectious Diseases Survey) studies to compare contact details from COVIMOD to pre-pandemic contact patterns. POLYMOD was a Europe-wide paper-based contact survey, conducted in Germany in 2005/2006. HaBIDS was conducted between 2014 and 2017 and collected data on social contacts of people living in certain districts of Lower Saxony, Germany, using both online and paper-based surveys. Participants in POLYMOD and HaBIDS were given the option to report group contacts if they were too numerous to list individually; however, this option was only provided for professional contacts. Further details on HaBIDS and POLYMOD can be found elsewhere [11, 12].

### Definition of the risk groups

We considered two groups of participants at higher risk of a severe COVID-19 outcome. First, all participants aged 65 and over were considered to be at increased risk due to their age. This risk will henceforth be called high ‘*age risk’* and correspondingly participants aged below 65 will be called the low *age risk* group.

The second group consisted of participants with underlying health conditions that risk a severe course of COVID-19. During the first 13 COVIMOD survey waves, participants were asked if they belonged to a risk group for whom annual flu vaccinations are recommended (due to a health condition). This included persons with chronic respiratory disease, chronic heart disease, chronic kidney disease, chronic liver disease, chronic neurological disease, diabetes (all types), immunosuppression (disease- or treatment-related), asplenia or splenic dysfunction, grade III obesity (BMI ≥ 40), and pregnant women (Additional File 1). Throughout the manuscript we will refer to this as high risk due to underlying health conditions, or, when a shorter term is needed, as simply high ‘*health risk’*.

From survey wave 14 onwards, this question was rephrased and asked if the participant was at either high or medium risk for a severe COVID-19 outcome due to existing health conditions. Medium risk was defined as including one or more of the following: less severe lung disease (such as asthma, COPD, emphysema, or bronchitis), heart disease (e.g. heart failure), diabetes, chronic kidney or liver disease, brain or nerve disease (such as Parkinson's disease, motor neuron disease, multiple sclerosis, or cerebral palsy), increased risk for infection, taking medications that can affect the immune system (such as low-dose steroids), BMI of 40 or more, pregnancy. High risk was defined as including one or more of the following: organ transplant, bone marrow or stem cell transplant in the past six months, current cancer treatment, blood or bone marrow cancer, severe lung disease (such as cystic fibrosis, severe asthma, or severe COPD), a condition that increases the risk of infection (such as SCID or sickle cell disease), immunosuppressive medications or high dose steroids, pregnancy with a severe heart disease. For this study, we considered high and medium risk to be equivalent under the umbrella of high risk due to underlying conditions.

### Handling missing data

Data about *age risk* (i.e. data about the participant’s age or age group) was missing in 0.05% of COVIMOD responses, whereas data about *health risk* was missing in 8% of COVIMOD responses, due to them not knowing or not being willing to give an answer to the relevant questions. There were no missing data in the outcome variables of daily number of contacts.

For the *age risk* analysis, we decided to use a complete case analysis approach due to the very low proportion of missing data.

It was often the case that data about participants’ *health risk* were missing from some survey waves but not others that they had also participated in. In this case, for each survey wave in which a participant had missing *health risk* status, we assigned them the *health risk* status from the temporally closest survey wave for which this information was not missing. After this procedure, we were left with 0.7% of responses with missing *health risk* status (due to it being missing from all relevant waves), and we removed these responses from the *health risk* analysis.

Since *health risk* status could change over time, we also did a sensitivity analysis for the *health risk* analysis, using an approach where we removed missing data in any survey wave completely regardless of whether it was available in another survey wave, instead of using the procedure described above.

### Data management and statistical analysis

Throughout this section, the participant refers to the person whose contacts were reported. All analyses were done using R version 4.1.1 [13] and Python 3.9.7 [14]. Participants in COVIMOD were asked to specify whether the contacts they reported were with household members or not. The analyses were carried out twice, once using all reported contacts, and once using only contacts with non-household members, which could possibly be more useful for assessing the impact of contact reduction measures. To prevent a few outliers with a large number of contacts from affecting the analyses, we decided to truncate the number of group contacts reported in each setting at 100 where necessary.

For COVIMOD we calculated the bootstrapped mean and 95% confidence intervals of the number of contacts per participant per survey wave split by risk status for participants stratified by both types of risk. To assess potential differences in contact patterns depending on risk status, we used negative binomial models with contacts as the independent variable, risk status and confounders (varied according to the analyses being performed) as explanatory variables.

In R notation:

$$contacts\sim risk\_status+confounders$$

In mathematical notation:

$$Contact{s}_{i}\sim NegativeBinomial\left(mean={\mu }_{i},heterogeneity=\alpha \right)$$

$$ln\left({\mu }_{i}\right)={\beta }_{0}+{\beta }_{1}{1}_{risk}\left(i\right)+{\sum }_{x}{\beta }_{x}{X}_{xi}+ {\epsilon }_{i}$$

where $i$ represents an individual participant, $j$ represents survey wave, ${1}_{risk}$ is an indicator function for whether participant $i$ belongs to the high risk group, and ${X}_{x}$ are confounders (varied according to the analyses being performed). The ${\beta }$ are coefficients to be estimated, and ${\epsilon }_{ij}$ is the residual term.

We fit the models to our data using generalised estimating equations (GEEs), to obtain population-averaged coefficients while taking into account autocorrelation due to repeated measures of the same participant. The exponentiated coefficient ${e}^{{\beta }_{1}}$ then represents the population-averaged ratio of mean contacts in the high risk group to the low risk group. Thus, we will henceforth call it the overall ‘contact ratio’ for high risk. We used the ‘statsmodels’ package in Python [15] to fit the models.

We used two ‘types’ of model in the analyses. Model 1 for both *age risk* and *health risk* included no confounding variables, and we will refer to the contact ratios obtained from it as ‘unadjusted’ contact ratios. For model 2 for *age risk*, we included sex and household size as confounding variables, since older people tend to live in smaller households than younger people. Similarly, certain age groups are more likely to be at high *health risk* than others, so we included sex and age group as confounding variables in model 2 for *health risk.* The contact ratios obtained from Model 2 will be called the ‘adjusted’ contact ratios.

#### Lifting of restrictions

The different COVIMOD survey waves took place under varying levels of contact restrictions in Germany. We identified five distinct ‘restriction phases’ in the duration of the analysed survey waves, based on chronologies of national-level restrictions in Germany [1, 16].

*First lockdown*: COVIMOD waves 1 and 2 were conducted towards the tail end of the first lockdown in Germany, in the end of April and most of May 2020.

*Relaxed measures*: COVIMOD waves 3 to 12 were conducted when contact restriction measures were relaxed in Germany, from the end of May to the start of November 2020. This includes ‘transition’ periods at the start when restrictions were being eased gradually, and at the end when restrictions were slowly being put in place again as a response to the second wave of the SARS-CoV-2 pandemic.

*Second lockdown*: The second SARS-CoV-2 wave led to an increase in contact restrictions in Germany again, starting in November 2020 and eventually a second lockdown that lasted till mid-April 2021, although schools and some businesses were allowed to reopen from the beginning of March 2021. COVIMOD waves 13 to 20 were carried out during this period.

*Relaxed measures again*: Restrictions were relaxed again once the second lockdown was lifted, and remained relaxed until late August. COVIMOD waves 21 to 25 were conducted during this time.

*Vaccination/Testing checks*: COVIMOD waves 26 to 31 were carried out from September 2021, when the so-called 3G, 2G, and 2G + rules were put in place in much of Germany. These restricted large parts of public life to people unless they had:

- 3G: Been vaccinated, recently tested negative for, or recently recovered from a SARS-CoV-2 infection,
- 2G: Been vaccinated or recently recovered from a SARS-CoV-2 infection,
- 2G + : Been vaccinated or recently recovered, AND recently tested negative for SARS-CoV-2.

These phases are similar to, but not the same as, the classification made by the Robert Koch Institute (RKI) [17, 18]; the intensity of contact reduction measures was not the main consideration in the RKI’s classification.

To establish whether COVIMOD participants responded differently to strict contact restriction measures being lifted, we took an interrupted time series approach considering the lifting of lockdown as the interruption, using the negative binomial model schema below adapted from the framework described by Linden [19]:

In R notation:

$$contacts\sim ris{k}\_{status}*wave+confounders$$

In mathematical notation:

$$Contact{s}_{ij}\sim NegativeBinomial\left(mean={\mu }_{ij},heterogeneity=\alpha \right)$$

$$ln\left({\mu }_{ij}\right)={\beta }_{0}+{\beta }_{1}{1}_{risk}\left(i\right)+{\beta }_{2}j+{\beta }_{3}{1}_{relaxed}\left(j\right)+{\beta }_{4}{1}_{risk}\left(i\right)j+{\beta }_{5}{1}_{relaxed}\left(j\right){1}_{risk}\left(i\right)+{\beta }_{6}j{1}_{relaxed}\left(j\right)+{\beta }_{7}j{1}_{relaxed}\left(j\right){1}_{risk}\left(i\right) {+{\sum }_{x}{{\beta }_{x}X}_{xi}+\epsilon }_{ij}$$

where the symbols mean the same as before, with the new indicator function ${1}_{relaxed}\left(j\right)$ indicating whether wave $j$ occurred during a time of relaxed restriction measures. We excluded waves belonging to the ‘*Vaccination/Testing checks’* phase (waves 26 onwards) from this analysis, since the restrictions during these waves were neither at strict lockdown levels nor fully relaxed.

A detailed interpretation of the regression coefficients from the above model schema can be found in the paper by Linden [19]; here we are only concerned with ${\beta }_{5}$. ${e}^{{\beta }_{5}}$ represents the (multiplicative) change in the contact ratio for high risk from the lockdown phases to the relaxed measures phases i.e. it is a ratio of contact ratios.

For this analysis too, we used two models for both the *age risk* and *health risk* analysis; Time series 1 with no confounders, and Time series 2 with the confounders mentioned earlier for Model 2. These models were also fit using GEEs to account for within-participant clustering.

#### Comparison to POLYMOD and HaBIDS

In POLYMOD, no information on underlying health conditions was collected, so POLYMOD was used only for the *age risk* comparison with COVIMOD. Similarly, in HaBIDS no participants older than 75 years were recruited, therefore HaBIDS was only included in the *health risk* comparison. For this purpose, we restricted COVIMOD participants to those between 15 and 75 years of age to match HaBIDS. The high *health risk* group in HaBIDS is made up of those participants who reported having a chronic condition, or currently being pregnant [12], with participants for whom this data was missing being excluded. Furthermore, due to different definitions of group contacts in the surveys, it was not possible to truncate contacts from all three studies in the same way, so we chose to right-truncate the final number of contacts at 100 for the comparative analysis.

The sample sizes for our HaBIDS (*N* = 856) and POLYMOD (*N* = 1341) datasets were similar to that of one COVIMOD wave (average *N* = 1761.4, min. 739, max. 2502) and did not have repeated measures. Therefore, to estimate contact ratios for high risk for these surveys, we used the models used for COVIMOD but without accounting for within-participant clustering. We used regression analyses to fit these models, using the ‘glm’ function from the MASS package in R [20]. Again, the 2-model framework was used to estimate both unadjusted and adjusted contact ratios for HaBIDS and POLYMOD separately. We compared these ratios to the overall contact ratios obtained from fitting the respective COVIMOD models to the modified COVIMOD data.

Neither POLYMOD nor HaBIDS required their participants to specify whether their contacts were with household members or not, so an analysis concerning contacts only with non-household members was not possible.

It has been reported that the day of the week, especially whether it is a weekend or a weekday, is a contributing factor to social contact behaviour, and it is common to weight contact survey data by day of reporting [21]. However, we found in descriptive analyses that the distribution of responses with respect to weekday or weekend was balanced in the high and low risk groups for both age and health risk (between 20 and 22% of responses made on a weekend in all groups), so we did not adjust or weight for this in our analyses. Furthermore, although we had data on whether COVIMOD participants were vaccinated in the survey waves conducted after vaccinations became available (survey wave 17 onwards), we believe that being unvaccinated due to vaccinations not being available would have a different impact on contact patterns than being unvaccinated when vaccinations were available, due to choice or vaccination prioritisation policies. Therefore, we did not find it appropriate to adjust for vaccination status.

## Results

### Demography

The first 31 COVIMOD survey waves had a total of 54,602 responses from 7,323 unique participants, who reported a total of 178,916 contacts including truncated group contacts. Of these, 54,570 responses were included in the *age risk* analysis, and 54,208 were included in the *health risk* analysis, with the others excluded due to missing information about their risk status. From POLYMOD, 1,341 participants, who reported a total of 24,278 contacts after truncation, were assessed in this study, as well as 856 participants from the HaBIDS study who reported a total of 27,777 contacts. All of the included HaBIDS participants were from the online-based part of the survey.

Between 43 and 49% of the responses in the COVIMOD waves were from female participants. Between 18 and 32% were from participants at high risk for a severe COVID-19 outcome due to their age (*age risk)* and between 35 and 40% were from participants at risk for severe COVID-19 due to underlying health conditions (*health risk)*. A detailed demographic table for COVIMOD can be found in Additional File 2, sheet 2. In comparison, 10% of POLYMOD participants were at high risk due to their age, and 15% of the participants analysed from HaBIDS were at high risk due to underlying health conditions.

### Mean contacts

Across all COVIMOD waves, participants reported a mean of 3.28 overall daily contacts (with group contacts truncated, bootstrapped 95%CI 3.20, 3.35). Participants at high *health risk* reported a mean of 2.87 (95%CI 2.77, 2.98) daily contacts, whereas those at low *health risk* reported a mean of 3.49 (95%CI 3.38, 3.60) daily contacts. Participants at high and low *age risk* reported a mean of 2.59 (95%CI 2.49, 2.70) and 3.52 (95%CI 3.43, 3.62) daily contacts respectively.

**Fig. 1:**
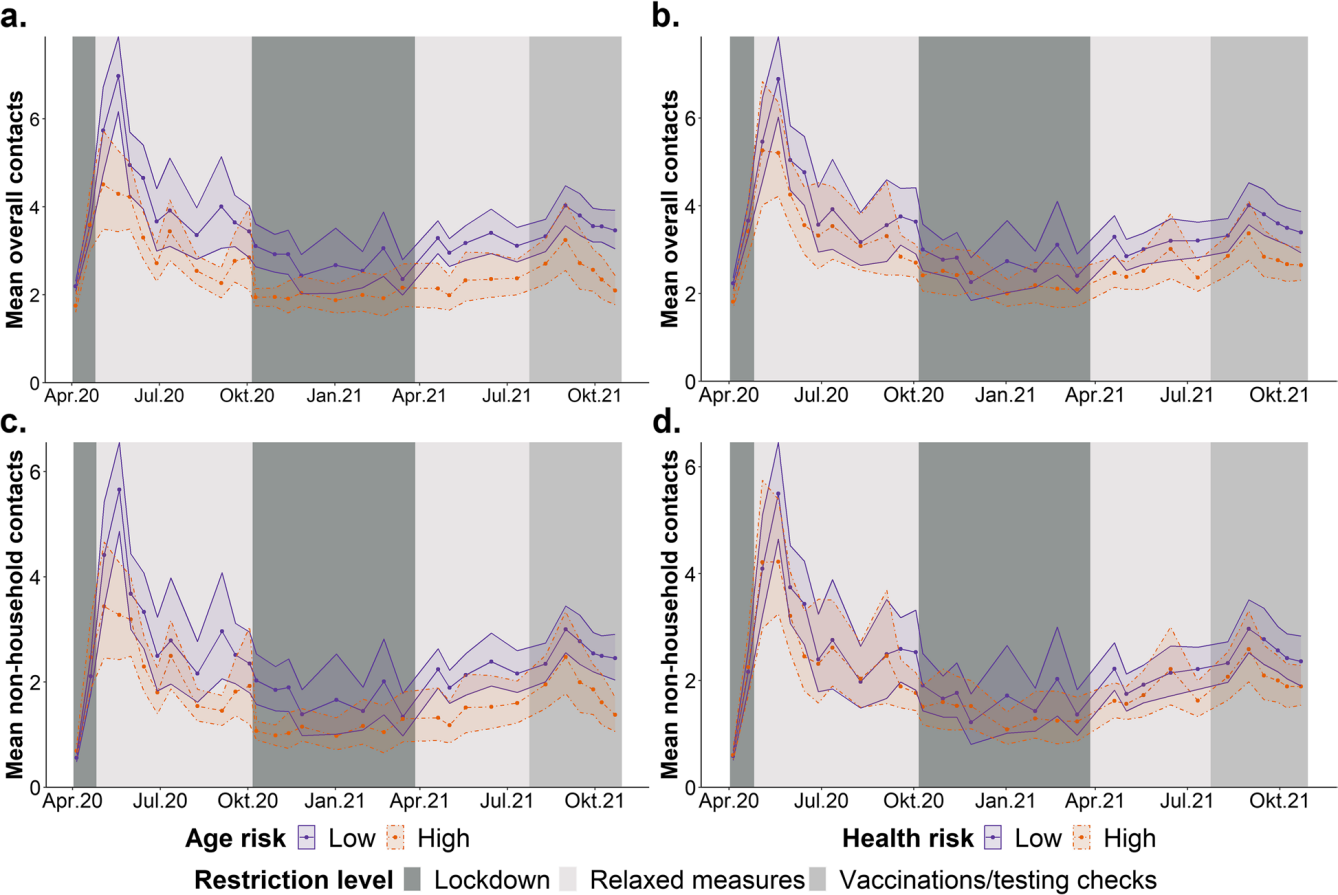
Mean number of contacts reported by participants per COVIMOD wave, split by risk status. 95% confidence intervals were obtained by bootstrapping. The x-axis represents midpoints of the period of data collection for the corresponding survey wave. Displayed are the mean overall number of contacts stratified by (**a**) whether the participant is aged over or under 65 (high or low age risk), and (**b**) whether the participant is recommended to get an annual flu vaccination due to existing health conditions or not (high or low health risk). The bottom row displays the mean number of contacts with non-household members, stratified by (**c**) age risk and (**d**) health risk status

Figure 1 below, and Tables A3.1 and A3.2 in Additional File 3 show the mean number of overall contacts and the mean number of contacts with non-household members stratified by risk group for each COVIMOD survey wave. In most waves, participants at high risk reported fewer contacts on average than participants at low risk. The difference was greatest in survey waves 4 and 5, i.e. June 2020, which happened during a period of relaxed contact restriction measures in Germany.

### Contact ratios

Table 1 shows both the unadjusted and adjusted overall contact ratios (CRs) for high risk for COVIMOD participants. In the unadjusted model, higher risk was associated with having fewer contacts, for both *age risk* and *health risk* (All contact CRs 0.73 [95%CI 0.66,0.81] for *age risk*, 0.82 [95%CI 0.75, 0.90] for *health risk,* non-household contact CRs 0.72 [95%CI 0.62,0.83] and 0.84 [95%CI 0.74,0.96] for *age* and *health* risk respectively*).* So, for example, the unadjusted ratios imply that COVIMOD participants aged 65 and over reported on average 27% fewer daily contacts than those younger than 65. The association with high *age risk* remained after adjusting for participant sex and household size (all contact CR 0.83 [95%CI 0.74,0.92], non-household contact CR 0.72 [95%CI 0.62,0.83]), but the association with high *health risk* disappeared after adjusting for participant sex and age group (all contact CR 0.99 [95%CI 0.90,1.09], non-household contact CR 0.97 [95%CI 0.86,1.10]).

**Table 1:** Overall contact ratios for high risk during the pandemic

	**Unadjusted contact ratio (95% CI)**	**Adjusted contact ratio (95% CI)**
*Health risk*		
All contacts	0.82 (0.75, 0.90)	0.99 (0.90, 1.09)
Non-household contacts	0.84 (0.74, 0.96)	0.97 (0.86, 1.10)
*Age risk*		
All contacts	0.73 (0.66, 0.81)	0.83 (0.74, 0.92)
Non-household contacts	0.72 (0.62, 0.83)	0.72 (0.62, 0.83)

Table 1: The unadjusted contact ratios were obtained by a model with risk status as the explanatory variable, accounting for clustering due to repeated measures of the same participant.. The adjusted contact ratios were obtained by adding sex and household size as confounders for the *age risk* analysis, and sex and age group for the *health risk* analysis

### Response to lifting restrictions

COVIMOD participants reported a mean of 2.60 (95%CI 2.48, 2.70) daily total contacts and 1.50 (95%CI 1.39, 1.61) daily non-household contacts in waves that occurred during the two lockdown phases, compared to a mean of 3.62 (95%CI 3.50, 3.75) and 2.56 (95%CI 2.44, 2.68) daily total and non-household contacts respectively in waves that occurred during the two ‘relaxed measures’ phases.

Table 2 shows the estimated change in contact ratios for high risk from lockdown phases to relaxed phases. The unadjusted contact ratios for high *age risk* with respect to non-household contacts were smaller during periods of relaxed measures than during lockdown periods (Ratio of CRs 0.72 [95%CI 0.54,0.95]). This was also true but with wide confidence intervals spanning either side of 1 for the contact ratios regarding all contacts (Ratio of CRs 0.87 [95%CI 0.72,1.05]). This remained the case after adjusting for sex and household size (Multiplicative factor 0.83 [95%CI 0.71,1.05] for all contacts, 0.72 [95%CI 0.54,0.95] for non-household contacts).

**Table 2:** Change in contact ratios for high risk due to lifting of restrictions

	**Change in contact ratio (Multiplicative) Unadjusted**	**Change in contact ratio (Multiplicative) Adjusted**
All contacts		
*Health risk*	0.92 (0.75, 1.13)	0.92 (0.76, 1.13)
*Age risk*	0.87 (0.72, 1.05)	0.83 (0.71, 1.05)
Non-Household contacts		
*Health risk*	0.79 (0.58, 1.07)	0.80 (0.60, 1.08)
*Age risk*	0.72 (0.54, 0.95)	0.72 (0.54, 0.95)

Table 2: The estimates were obtained by an interrupted time series analysis as described in the methods. The unadjusted estimates are from a model without adjusting for confounding variables, whereas the adjusted estimates were obtained by adjusting for sex and household size in the age risk analysis, and sex and age group in the health risk analysis

The point estimates for the ratio of contact ratios for high *health risk* pointed towards lower contact ratios during periods of relaxed measure compared to lockdown periods, but confidence intervals were wide and spanning either side of 1 (Ratio of CRs 0.92 [95%CI 0.75,1.13] for all contacts, 0.79 [95%CI 0.58,1.07] for non-household contacts). This remained true after adjusting for sex and age group (Ratio of CRs 0.92 [95%CI 0.76,1.13] and 0.80 [95%CI 0.60,1.08] for all and non-household contacts respectively).

### Comparison to POLYMOD and HaBIDS

116 responses from COVIMOD (0.2%) required further truncation of grouped contacts to make them suitable for comparison compared to 49 HaBIDS responses (5%), and 2 POLYMOD responses (0.1%).

For POLYMOD, after this truncation, people at high *age risk* reported a mean of 7.00 (95%CI 5.62,8.52) contacts, compared to 19.55 (95%CI 18.51,20.54) contacts for those at low risk. Comparatively, after this truncation, the mean contacts for high and low *age risk* in COVIMOD were 2.56 (95%CI 2.47,2.66) and 3.44 (95%CI 3.36,3.52) respectively.

For comparison to HaBIDS, after excluding COVIMOD participants outside the HaBIDS age range of 16–75 years and those for whom no data about *health risk* were available, we were left with 48,630 COVIMOD responses. Of these, 48% were from female participants and 37% from participants at increased risk due to underlying health conditions (*health risk* group).

In HaBIDS, the mean number of contacts reported by people with an underlying health condition (*health risk* group) was 26.49 (95%CI 21.65,31.80), compared to 28.58 (95%CI 26.71,30.42) for those at low risk. In COVIMOD on the other hand, the high *health risk* group (in HaBIDS-compatible age groups) reported a mean of 2.82 (95%CI 2.72,2.93) contacts, compared to 3.20 (95%CI 3.11, 3.30) for the low *health risk* group.

For the sake of completeness, the mean reported contacts and bootstrapped 95% confidence intervals by risk status for each COVIMOD wave after this truncation, and after further filtration for compatible age groups, can be found in Additional File 3, Table A3.3. Aside from a slight reduction in the numbers due to group contacts being truncated to smaller values, these means are not much different from the ones in Fig. 1 in the ‘Mean Contacts’ section above, and in Additional File 3 Tables A3.1 and A3.2.

Table 3 shows the estimated contact ratios for high risk status for POLYMOD, HaBIDS, and the overall contact ratios for the modified-for-comparison COVIMOD data, without and with further adjusting variables respectively. Both the unadjusted and adjusted (for sex and household size) models showed that high *age risk* was associated with fewer contacts in POLYMOD (Unadjusted CR 0.36 [95%CI 0.30,0.42], adjusted CR 0.36 [95%CI 0.30,0.43]). This effect was much stronger than that observed in the comparable COVIMOD data (Unadjusted CR 0.74 [95%CI 0.68,0.82], adjusted CR 0.84 [95%CI 0.76,0.92]).

**Table 3:** Comparison of contact ratios for high risk before and during the pandemic

	**Unadjusted Contact Ratio (95%CI)**	**Adjusted Contact Ratio (95%CI)**
*Health risk*		
Pre-pandemic (HaBIDS)	0.93 (0.77, 1.13)	0.96 (0.80, 1.16)
Pandemic (COVIMOD)	0.88 (0.80, 0.97)	0.99 (0.91, 1.09)
*Age risk*		
Pre-pandemic (POLYMOD)	0.36 (0.30, 0.42)	0.36 (0.30, 0.43)
Pandemic (COVIMOD)	0.74 (0.68, 0.82)	0.84 (0.76,0.92)

Table 3: Contacts were right-truncated at 100 for this analysis. COVIMOD estimates were obtained by running the same models as before on the dataset modified to be compatible with HaBIDS and POLYMOD. Pre-pandemic estimates were obtained by a negative binomial regression analysis. When other adjusting variables were used, they were sex and household size for the *age risk* analysis, and sex and age group for the *health risk* analysis

In HaBIDS, neither the unadjusted nor the adjusted (for sex and age group) contact ratios showed an association between high *health risk* and differences in contact behaviour, but the confidence intervals were wide (Unadjusted CR 0.93 [95%CI 0.77,1.13], adjusted CR 0.96 [95%CI 0.80,1.16]). In the comparable COVIMOD dataset, the unadjusted analysis showed an association between high *health risk* and fewer contacts (CR 0.88 [95%CI 0.80,0.97]), which disappeared after adjusting for sex and age group (CR 0.99 [95%CI 0.91,1.09]).

### Sensitivity analysis for health risk

We found that using an approach where we removed missing data from each survey wave regardless of whether it was available in other waves, instead of the ‘temporal imputation’ method described in the methods section, to handle missing data about *health risk* led to slight changes in the values of the mean contacts and contact ratios (in particular, mean contacts were higher for both risk groups in the complete case analysis). However, these differences were small and did not impact the interpretation of the results at all.

## Discussion

Reductions in the contacts of the general population caused by the pandemic and implemented control measures have been extensively studied [3–7, 22, 23]. The COVIMOD survey also shows that there has been a reduction in contacts throughout the German population during the pandemic, and in this study we further found that for most of the duration of the pandemic, people with high risk status had on average fewer contacts than people at lower risk (See Fig. 1, Additional File 3 tables A3.1 and A3.2).

It is already well-documented that elderly persons had fewer contacts than younger people, even before the pandemic [24, 25]. The pre-pandemic data we analysed from the POLYMOD survey corroborates this, and data from COVIMOD shows that this phenomenon remained during the pandemic, with elderly participants reporting fewer contacts than younger participants.

Compared to pre-pandemic times, the difference in pandemic contact patterns between the high and low *age risk* groups (i.e. Respectively the elderly and younger age groups) was less stark. A possible explanation for this could be that the contact reduction measures employed during the pandemic have mainly been successful at reducing contacts in work and education settings, which reduces the younger population’s contacts disproportionately more than elderly people’s contacts. This can be seen from the POLYMOD and COVIMOD data; the work contacts of the elderly remain consistently low in both POLYMOD and all COVIMOD waves, but the work contacts of the younger individuals are much lower in the COVIMOD waves than in POLYMOD (Additional File 3 Table A3.4). Another possible explanation is that there was simply less scope for reduction in the elderly’s contacts, since they had much fewer contacts to begin with. Whatever the reason, this has implications for the parametrisation of infectious disease models. Many mathematical models of the pandemic were parametrised using scaled-down pre-pandemic data [26–28]. Given the differences in age-stratified contact patterns betweeen pre-pandemic and pandemic times, such a uniform scaling down may not be appropriate.

We found that participants at risk because of underlying health conditions (*health risk*) also reported fewer contacts during the pandemic compared to participants without this risk, as can be seen from Fig. 1 and the unadjusted contact ratios in Table 1. However, the magnitude of this effect was lower compared to that due to *age risk,* as illustrated in Table 1. Furthermore, given that, after adjusting for age and sex, the effect disappeared, there is no evidence that this reduction is associated with high *health risk* itself*,* and it is likely associated with the confounding effect of age instead.

We did not find a strong association between *health risk* status and differences in contact behaviour in pre-pandemic data from HaBIDS, in either the unadjusted or adjusted analyses. Although we did find such an association in the unadjusted analysis in COVIMOD, the overlapping confidence intervals mean that it is unclear whether there were changes in this effect from pre-pandemic to pandemic times.

We also found evidence of differential responses to the lifting of strict contact restriction measures (i.e. lockdown) based on *age risk* status. The average number of contacts of both the high and low risk groups increased in the survey waves immediately after restrictions were lifted or relaxed (Fig. 1), but the high *age risk* group increased their non-household contacts less than the low *age risk* group (as indicated by the change in contact ratios in Table 3). The picture for *health risk* is, once again, less clear, as is the picture for *age risk* when considering all contacts; the wide confidence intervals point, at best, to weak evidence to the presence of a differential response in these cases. Further study is needed to understand if people reacted differently to the lifting of strict contact restriction measures based on individual risk status.

Although various quotas were put in place for recruitment to the COVIMOD participant pool to ensure that the distribution of sociodemographic characteristics matched that of the German population as closely as possible, middle aged individuals with young children were underrepresented because many such individuals took up the option of filling in the survey as a proxy for their children, instead of with their own information. Furthermore, it cannot be ruled out that the people inclined to take part in a survey relating to public health would also be more inclined to adhere more closely to infection prevention measures, and this is a potential source of bias. Lastly, the participant pool might not have been representative of the German population with regards to epidemiological characteristics even if it was representative with regards to sociodemographic ones.

Details about other sources of bias in COVIMOD, for example survey fatigue from participants taking part in multiple waves, and what steps were taken to mitigate these biases, can be found elsewhere [7]. The COVIMOD questionnaire also changed over the 31 waves, the most prominent change being the addition of group contacts from survey wave 3 onwards. The lower contacts seen in the first two survey waves could be an artefact of this change. Although we cannot know for sure, it is likely that the inability to give group contacts would affect the group that would have a higher number of contacts disproportionately more than the other. This would mean that we would underestimate the magnitude of any differences in contact behaviour in these waves. Given that this was only the case for the first 2 waves, however, it likely had little to no impact on the overall estimates.

All 3 surveys analysed in this paper were held in differing time periods and had different participant pools. Furthermore, they had very different sample sizes and used different means of data collection—POLYMOD was paper-based, with participants encouraged to carry the booklets with them and record contacts as they happened, HaBIDS was a mix of online and paper-based data collection although only participants from the online survey were analysed in this study, and COVIMOD was completely online. There is evidence to show that contact reporting remains consistent between online and mixed-mode surveys [29], but participants are likely to report more contacts in paper-based surveys than online ones [30]. It should be noted that this is contrary to our pre-pandemic data; the analysed POLYMOD participants reported on average around 18 contacts, while the analysed HaBIDS participants reported an average of around 32. This is unlikely to have an effect on comparisons of reported contact behaviour of different groups within a single survey, but makes cross-survey comparisons less clear. In addition, the group contacts assessed in POLYMOD (as well as HaBIDS) only include contacts met at work. Since the elderly are less likely to have a lot of work contacts, it cannot be ruled out that the difference in the number of contacts of older participants compared to younger participants in POLYMOD has been overestimated. Furthermore, the definitions of *health risk* in HaBIDS and COVIMOD are not an exact match; therefore, the results regarding persons with underlying health conditions should be interpreted with caution.

## Conclusion

We found that people at higher risk of developing a severe outcome from a SARS-CoV-2infection due to age or an underlying health condition had, *on a population level*, fewer daily contacts during the pandemic than people at lower risk. Interestingly, the difference in the number of contacts between people at higher risk due to old age and those not was smaller during the pandemic than before. Furthermore, persons at higher risk due to their age increased their contacts less than those not when contact restriction measures were lifted. These differences should be taken into account when parametrising infectious disease models in pandemic settings. However, the population-level differences observed within the subpopulation at higher risk due to underlying health conditions compared to those at lower risk were likely due to other factors and had no association with risk status itself.

## Supplementary Information

**Additional file 1.** COVIMOD Questionnaire. This file contains the questionnaire used for survey wave 4 of the COVIMOD study.

**Additional file 2.** Demography and survey wave details. This file contains dates and sample sizes for each COVIMOD survey wave, as well as a demography table.

**Additional file 3.** Tabular and additional results. This file contains the numerical results shown as figures in the manuscript, and some additional results.

## Data Availability

The datasets used and/or analysed during the current study are available from the corresponding author on reasonable request.
