# Supplementary material for: Effect of risk status for severe COVID-19 on individual contact behaviour during the SARS-CoV-2 pandemic in 2020/2021—an analysis based on the German COVIMOD study
Source: BMC Infect Dis. 2023 Apr 6;23:205. doi: 10.1186/s12879-023-08175-2 (PMC10078023; doi:10.1186/s12879-023-08175-2)
Supplement: Supplementary file 3 — Additional file 3. Tabular and additional results. This file contains the numerical results shown as figures in the manuscript, and some additional results. [file 12879_2023_8175_MOESM3_ESM.docx]

**Effect of risk status for severe COVID*-*19 on individual contact behaviour during the SARS-CoV-2 pandemic in 2020/2021 - an analysis based on the German COVIMOD study**

**Additional File 3: Per-wave tabular results of the analyses conducted**

Jasmin Walde^1*^, Madhav Chaturvedi^1*^, Tom Berger^1^, Antonia Bartz^1^, Robin Killewald^1^, Damilola Victoria Tomori^1^, Nicole Rübsamen^1^, Berit Lange^2,3^, Stefan Scholz^4^, Marina Treskova^4,5^, Karolin Bucksch^6^, Christopher I Jarvis^7^, Rafael Mikolajczyk^8^, André Karch^1^, Veronika K Jaeger^1^

^1^ Institute of Epidemiology and Social Medicine, University of Münster, Münster, Germany

^2^ Department of Epidemiology, Helmholtz Centre for Infection Research, Braunschweig, Germany

^3^ German Center for Infection Research, Braunschweig, Germany

^4^ Immunization Unit, Infectious disease epidemiology, Robert Koch-Institute, Berlin, Germany

^5^Heidelberg Institute of Global Health, Heidelberg University Hospital, Heidelberg, Germany

^6^Institute for Medical Informatics, Statistics and Epidemiology (IMISE), University of Leipzig, Leipzig, Germany

^7^London School of Hygiene and Tropical Medicine, London, United Kingdom

^8^Institute for Medical Epidemiology, Biostatistics and Informatics, University of Halle, Halle, Germany

*contributed equally as first authors

**Corresponding author**

**Table A3.1**: Mean and bootstrapped 95% confidence intervals for both all and only non-household contacts for each COVIMOD survey wave, split by whether the participant is aged over 65 or not (high or low *age risk*).

|  | All Contacts (95%CI) | | Non-household contacts (95%CI) | |
| --- | --- | --- | --- | --- |
| Survey Wave | **Low risk:**  **Mean (95% CI)** | **High risk:**  **Mean (95% CI)** | **Low risk:**  **Mean (95% CI)** | **High risk:**  **Mean (95% CI)** |
| 01: 30.04. – 06.05.2020 | 2.19 (2.08,2.31) | 1.75 (1.6,1.92) | 0.56 (0.48,0.64) | 0.69 (0.57,0.82) |
| 02: 14.05. – 21.05.2020 | 3.57 (3.24,3.98) | 3.59 (2.99,4.37) | 2.11 (1.78,2.5) | 2.47 (1.87,3.22) |
| 03: 28.05. – 04.06.2020 | 5.73 (4.75,6.72) | 4.51 (3.5,5.73) | 4.42 (3.43,5.43) | 3.44 (2.46,4.66) |
| 04: 11.06. – 22.06.2020 | 6.97 (6.16,7.87) | 4.29 (3.44,5.28) | 5.66 (4.86,6.55) | 3.28 (2.42,4.27) |
| 05: 26.06. – 01.07.2020 | 4.95 (4.26,5.69) | 4.22 (3.5,5.01) | 3.68 (3,4.44) | 3.19 (2.49,4) |
| 06: 09.07. – 16.07.2020 | 4.66 (3.95,5.41) | 3.3 (2.81,3.88) | 3.33 (2.63,4.07) | 2.29 (1.82,2.86) |
| 07: 24.07. – 29.07.2020 | 3.67 (2.99,4.41) | 2.72 (2.31,3.18) | 2.49 (1.83,3.23) | 1.8 (1.4,2.27) |
| 08: 07.08. – 11.08.2020 | 3.92 (3.11,5.11) | 3.44 (2.78,4.16) | 2.79 (1.96,3.98) | 2.5 (1.87,3.17) |
| 09: 04.09. – 09.09.2020 | 3.36 (2.81,3.98) | 2.54 (2.23,2.89) | 2.16 (1.62,2.77) | 1.54 (1.26,1.86) |
| 10: 30.09. – 05.10.2020 | 4.01 (3.06,5.14) | 2.27 (1.93,2.62) | 2.96 (2.06,4.08) | 1.45 (1.18,1.75) |
| 11: 14.10. – 21.10.2020 | 3.65 (3.1,4.27) | 2.77 (2.29,3.4) | 2.51 (1.97,3.12) | 1.82 (1.36,2.45) |
| 12: 29.10. – 03.11.2020 | 3.44 (2.85,4.03) | 2.85 (2.11,3.97) | 2.35 (1.79,2.95) | 1.93 (1.2,3.05) |
| 13: 05.11. – 10.11.2020 | 3.11 (2.64,3.61) | 1.95 (1.75,2.14) | 2.03 (1.57,2.53) | 1.07 (0.9,1.26) |
| 14: 25.11. – 30.11.2020 | 2.92 (2.51,3.37) | 1.95 (1.74,2.16) | 1.85 (1.46,2.29) | 0.98 (0.8,1.18) |
| 15: 09.12. – 15.12.2020 | 2.92 (2.46,3.47) | 1.91 (1.59,2.31) | 1.9 (1.44,2.44) | 1.03 (0.74,1.42) |
| 16: 23.12. – 30.12.2020 | 2.44 (2.02,2.92) | 2.04 (1.75,2.42) | 1.39 (0.98,1.86) | 1.15 (0.88,1.5) |
| 17: 28.01. – 02.02.2021 | 2.67 (2.04,3.52) | 1.88 (1.59,2.24) | 1.66 (1.01,2.53) | 0.98 (0.71,1.32) |
| 18: 24.02. – 03.03.2021 | 2.54 (2.16,2.98) | 1.99 (1.63,2.51) | 1.45 (1.09,1.9) | 1.16 (0.82,1.68) |
| 19: 17.03. – 26.03.2021 | 3.06 (2.42,3.88) | 1.92 (1.51,2.45) | 2.01 (1.38,2.82) | 1.05 (0.65,1.55) |
| 20: 07.04. – 15.04.2021 | 2.36 (1.99,2.8) | 2.16 (1.73,2.7) | 1.34 (0.98,1.79) | 1.3 (0.86,1.81) |
| 21: 12.05. – 24.05.2021 | 3.29 (2.94,3.68) | 2.14 (1.7,2.72) | 2.24 (1.89,2.63) | 1.32 (0.89,1.89) |
| 22: 26.05. – 03.06.2021 | 2.96 (2.65,3.28) | 1.99 (1.65,2.43) | 1.89 (1.6,2.22) | 1.18 (0.84,1.64) |
| 23: 09.06. – 22.06.2021 | 3.17 (2.78,3.57) | 2.33 (1.85,2.96) | 2.13 (1.75,2.54) | 1.51 (1.04,2.15) |
| 24: 07.07. – 19.07.2021 | 3.41 (2.94,3.95) | 2.36 (1.94,2.95) | 2.39 (1.92,2.93) | 1.53 (1.12,2.13) |
| 25: 04.08. – 13.08.2021 | 3.11 (2.75,3.53) | 2.38 (2,2.8) | 2.16 (1.8,2.59) | 1.6 (1.22,2.02) |
| 26: 01.09. – 14.09.2021 | 3.33 (2.98,3.72) | 2.71 (2.24,3.28) | 2.35 (2.01,2.74) | 1.96 (1.49,2.51) |
| 27: 22.09. – 06.10.2021 | 4.03 (3.57,4.48) | 3.24 (2.56,4.09) | 3 (2.56,3.44) | 2.48 (1.78,3.34) |
| 28: 08.10. – 20.10.2021 | 3.8 (3.35,4.3) | 2.72 (2.13,3.5) | 2.78 (2.34,3.27) | 2 (1.42,2.79) |
| 29: 22.10. – 02.11.2021 | 3.56 (3.2,3.97) | 2.57 (2.09,3.04) | 2.54 (2.19,2.94) | 1.86 (1.38,2.33) |
| 30: 03.11. – 09.11.2021 | 3.56 (3.2,3.94) | 2.34 (1.91,2.87) | 2.5 (2.14,2.88) | 1.61 (1.19,2.15) |
| 31: 17.11. – 23.11.2021 | 3.47 (3.05,3.92) | 2.09 (1.78,2.44) | 2.46 (2.04,2.91) | 1.38 (1.06,1.71) |

**Table A3.2**: Mean and bootstrapped 95% confidence intervals for both all and only non-household contacts for each COVIMOD survey wave, split by whether the participant is at high risk due to an underlying health condition or not (high or low *health risk*).

|  | All Contacts | | Non-household contacts | |
| --- | --- | --- | --- | --- |
| Survey Wave | **Low risk:**  **Mean (95% CI)** | **High risk:**  **Mean (95% CI)** | **Low risk:**  **Mean (95% CI)** | **High risk:**  **Mean (95% CI)** |
| 01: 30.04. – 06.05.2020 | 2.23 (2.11,2.36) | 1.81 (1.67,1.97) | 0.59 (0.5,0.68) | 0.6 (0.5,0.71) |
| 02: 14.05. – 21.05.2020 | 3.66 (3.3,4.07) | 3.42 (2.86,4.09) | 2.16 (1.83,2.57) | 2.25 (1.72,2.93) |
| 03: 28.05. – 04.06.2020 | 5.46 (4.56,6.47) | 5.26 (4.01,6.83) | 4.09 (3.21,5.09) | 4.21 (2.98,5.75) |
| 04: 11.06. – 22.06.2020 | 6.89 (6.03,7.86) | 5.21 (4.22,6.36) | 5.5 (4.65,6.45) | 4.22 (3.25,5.39) |
| 05: 26.06. – 01.07.2020 | 5.04 (4.36,5.83) | 4.26 (3.53,5.06) | 3.74 (3.07,4.53) | 3.21 (2.51,4.03) |
| 06: 09.07. – 16.07.2020 | 4.76 (4.01,5.58) | 3.56 (2.89,4.43) | 3.43 (2.67,4.23) | 2.45 (1.8,3.3) |
| 07: 24.07. – 29.07.2020 | 3.57 (2.95,4.42) | 3.32 (2.57,4.53) | 2.39 (1.79,3.24) | 2.31 (1.57,3.52) |
| 08: 07.08. – 11.08.2020 | 3.92 (3.01,5.06) | 3.54 (2.78,4.45) | 2.76 (1.84,3.89) | 2.61 (1.9,3.51) |
| 09: 04.09. – 09.09.2020 | 3.17 (2.64,3.8) | 3.09 (2.54,3.8) | 1.97 (1.49,2.56) | 2.04 (1.52,2.76) |
| 10: 30.09. – 05.10.2020 | 3.55 (2.73,4.57) | 3.31 (2.46,4.64) | 2.49 (1.68,3.49) | 2.47 (1.62,3.77) |
| 11: 14.10. – 21.10.2020 | 3.75 (3.1,4.52) | 2.84 (2.45,3.33) | 2.58 (1.94,3.32) | 1.89 (1.5,2.35) |
| 12: 29.10. – 03.11.2020 | 3.64 (2.96,4.37) | 2.7 (2.34,3.12) | 2.53 (1.85,3.25) | 1.76 (1.42,2.16) |
| 13: 05.11. – 10.11.2020 | 3 (2.53,3.56) | 2.41 (2.06,2.84) | 1.9 (1.43,2.46) | 1.51 (1.17,1.92) |
| 14: 25.11. – 30.11.2020 | 2.77 (2.41,3.22) | 2.51 (1.98,3.13) | 1.66 (1.32,2.08) | 1.6 (1.08,2.22) |
| 15: 09.12. – 15.12.2020 | 2.81 (2.33,3.39) | 2.42 (1.95,3.01) | 1.77 (1.31,2.33) | 1.53 (1.07,2.08) |
| 16: 23.12. – 30.12.2020 | 2.26 (1.84,2.82) | 2.47 (2.04,2.98) | 1.22 (0.8,1.75) | 1.52 (1.1,2) |
| 17: 28.01. – 02.02.2021 | 2.74 (2.02,3.66) | 2 (1.71,2.32) | 1.72 (1.01,2.65) | 1.08 (0.81,1.42) |
| 18: 24.02. – 03.03.2021 | 2.52 (2.14,2.96) | 2.19 (1.79,2.71) | 1.43 (1.05,1.86) | 1.29 (0.92,1.8) |
| 19: 17.03. – 26.03.2021 | 3.11 (2.43,4.1) | 2.11 (1.68,2.67) | 2.03 (1.34,3) | 1.25 (0.81,1.8) |
| 20: 07.04. – 15.04.2021 | 2.4 (2,2.9) | 2.09 (1.71,2.57) | 1.36 (0.98,1.83) | 1.23 (0.87,1.68) |
| 21: 12.05. – 24.05.2021 | 3.29 (2.87,3.78) | 2.47 (2.14,2.82) | 2.22 (1.82,2.71) | 1.62 (1.31,1.96) |
| 22: 26.05. – 03.06.2021 | 2.85 (2.53,3.24) | 2.38 (2.1,2.71) | 1.75 (1.43,2.12) | 1.56 (1.27,1.89) |
| 23: 09.06. – 22.06.2021 | 3.01 (2.66,3.38) | 2.52 (2.08,2.98) | 1.92 (1.58,2.29) | 1.73 (1.32,2.18) |
| 24: 07.07. – 19.07.2021 | 3.2 (2.75,3.71) | 3.01 (2.34,3.82) | 2.15 (1.71,2.65) | 2.21 (1.54,3.01) |
| 25: 04.08. – 13.08.2021 | 3.21 (2.82,3.63) | 2.36 (2.05,2.74) | 2.21 (1.83,2.63) | 1.62 (1.33,1.99) |
| 26: 01.09. – 14.09.2021 | 3.32 (2.95,3.71) | 2.86 (2.41,3.33) | 2.33 (1.97,2.73) | 2.07 (1.63,2.53) |
| 27: 22.09. – 06.10.2021 | 4.01 (3.57,4.53) | 3.37 (2.76,4.1) | 2.97 (2.52,3.51) | 2.58 (1.98,3.31) |
| 28: 08.10. – 20.10.2021 | 3.81 (3.33,4.37) | 2.84 (2.38,3.44) | 2.77 (2.3,3.35) | 2.09 (1.64,2.66) |
| 29: 22.10. – 02.11.2021 | 3.6 (3.19,4.05) | 2.76 (2.34,3.22) | 2.56 (2.16,3.01) | 2 (1.59,2.46) |
| 30: 03.11. – 09.11.2021 | 3.5 (3.11,3.96) | 2.67 (2.27,3.1) | 2.42 (2.03,2.87) | 1.89 (1.49,2.31) |
| 31: 17.11. – 23.11.2021 | 3.39 (2.93,3.87) | 2.65 (2.3,3.05) | 2.36 (1.9,2.83) | 1.89 (1.54,2.28) |

**Table A3.3**: Mean contacts (of all contacts) split by risk status in COVIMOD datasets used for comparison against POLYMOD and HaBIDS. Contacts were right truncated at 100; additionally, for the HaBIDS comparison, only participants between the ages of 15 and 75 were included. The risk status in question is *age risk* for the POLYMOD comparison and *health risk* for the HaBIDS comparison.

|  | COVIMOD dataset for POLYMOD comparison | | COVIMOD dataset for HaBIDS comparison | |
| --- | --- | --- | --- | --- |
| Survey Wave | **Low risk:**  **Mean (95% CI)** | **High risk:**  **Mean (95% CI)** | **Low risk:**  **Mean (95% CI)** | **High risk:**  **Mean (95% CI)** |
| 01: 30.04. – 06.05.2020 | 2.19 (2.08,2.31) | 1.75 (1.6,1.92) | 2.1 (1.97,2.23) | 1.8 (1.66,1.96) |
| 02: 14.05. – 21.05.2020 | 3.57 (3.24,3.97) | 3.59 (2.99,4.37) | 3.65 (3.27,4.08) | 3.49 (2.92,4.24) |
| 03: 28.05. – 04.06.2020 | 5.57 (4.68,6.42) | 4.51 (3.5,5.73) | 5.07 (4.24,6.01) | 5.12 (3.99,6.46) |
| 04: 11.06. – 22.06.2020 | 6.67 (5.97,7.46) | 4.21 (3.41,5.09) | 6.3 (5.49,7.18) | 4.9 (3.99,5.92) |
| 05: 26.06. – 01.07.2020 | 4.82 (4.19,5.46) | 4.22 (3.5,5.01) | 4.67 (4,5.35) | 4.27 (3.5,5.18) |
| 06: 09.07. – 16.07.2020 | 4.52 (3.88,5.2) | 3.3 (2.81,3.88) | 4.74 (4.02,5.53) | 3.48 (2.89,4.25) |
| 07: 24.07. – 29.07.2020 | 3.47 (2.93,4.04) | 2.72 (2.31,3.18) | 3.34 (2.79,3.97) | 3.18 (2.56,3.95) |
| 08: 07.08. – 11.08.2020 | 3.59 (3.01,4.29) | 3.44 (2.78,4.16) | 3.26 (2.69,3.95) | 3.56 (2.8,4.48) |
| 09: 04.09. – 09.09.2020 | 3.36 (2.81,3.98) | 2.54 (2.23,2.89) | 3.02 (2.39,3.73) | 3.12 (2.52,3.85) |
| 10: 30.09. – 05.10.2020 | 3.9 (3.05,4.92) | 2.27 (1.93,2.62) | 3.13 (2.42,3.92) | 3.38 (2.45,4.53) |
| 11: 14.10. – 21.10.2020 | 3.59 (3.06,4.16) | 2.76 (2.29,3.39) | 3.46 (2.85,4.12) | 2.84 (2.42,3.39) |
| 12: 29.10. – 03.11.2020 | 3.41 (2.83,3.96) | 2.65 (2.11,3.38) | 3.43 (2.78,4.18) | 2.66 (2.28,3.15) |
| 13: 05.11. – 10.11.2020 | 3.11 (2.64,3.61) | 1.95 (1.75,2.14) | 2.73 (2.28,3.25) | 2.44 (2.04,2.87) |
| 14: 25.11. – 30.11.2020 | 2.92 (2.51,3.37) | 1.95 (1.74,2.16) | 2.62 (2.2,3.09) | 2.52 (1.96,3.23) |
| 15: 09.12. – 15.12.2020 | 2.85 (2.45,3.3) | 1.91 (1.59,2.31) | 2.52 (2.1,3.02) | 2.44 (1.93,3.05) |
| 16: 23.12. – 30.12.2020 | 2.36 (2.01,2.75) | 2.04 (1.75,2.42) | 2.13 (1.77,2.57) | 2.43 (1.98,2.95) |
| 17: 28.01. – 02.02.2021 | 2.51 (2,3.12) | 1.88 (1.59,2.24) | 2.59 (1.91,3.38) | 1.94 (1.66,2.25) |
| 18: 24.02. – 03.03.2021 | 2.51 (2.16,2.91) | 1.99 (1.63,2.51) | 2.39 (2.02,2.85) | 2.19 (1.76,2.76) |
| 19: 17.03. – 26.03.2021 | 2.78 (2.36,3.26) | 1.92 (1.51,2.45) | 2.7 (2.24,3.26) | 2.08 (1.6,2.7) |
| 20: 07.04. – 15.04.2021 | 2.35 (1.99,2.77) | 2.16 (1.73,2.7) | 2.31 (1.87,2.87) | 2.1 (1.69,2.59) |
| 21: 12.05. – 24.05.2021 | 3.23 (2.91,3.56) | 2.13 (1.7,2.68) | 3 (2.61,3.43) | 2.4 (2.08,2.77) |
| 22: 26.05. – 03.06.2021 | 2.93 (2.64,3.24) | 1.99 (1.65,2.43) | 2.59 (2.23,2.99) | 2.33 (2.02,2.69) |
| 23: 09.06. – 22.06.2021 | 3.12 (2.75,3.48) | 2.25 (1.85,2.72) | 2.76 (2.4,3.15) | 2.48 (2.04,2.98) |
| 24: 07.07. – 19.07.2021 | 3.26 (2.87,3.69) | 2.29 (1.94,2.74) | 2.86 (2.47,3.31) | 2.88 (2.31,3.48) |
| 25: 04.08. – 13.08.2021 | 3.08 (2.73,3.48) | 2.38 (2,2.8) | 3.15 (2.73,3.59) | 2.4 (2.02,2.81) |
| 26: 01.09. – 14.09.2021 | 3.27 (2.97,3.62) | 2.71 (2.24,3.28) | 2.91 (2.58,3.28) | 2.81 (2.36,3.34) |
| 27: 22.09. – 06.10.2021 | 3.96 (3.54,4.34) | 3.1 (2.54,3.81) | 3.56 (3.15,4.01) | 3.09 (2.61,3.7) |
| 28: 08.10. – 20.10.2021 | 3.71 (3.32,4.13) | 2.58 (2.12,3.15) | 3.31 (2.87,3.75) | 2.73 (2.29,3.26) |
| 29: 22.10. – 02.11.2021 | 3.53 (3.18,3.91) | 2.55 (2.08,3) | 3.38 (2.94,3.88) | 2.72 (2.3,3.18) |
| 30: 03.11. – 09.11.2021 | 3.53 (3.18,3.91) | 2.34 (1.91,2.87) | 3.11 (2.7,3.56) | 2.73 (2.31,3.27) |
| 31: 17.11. – 23.11.2021 | 3.36 (3.01,3.73) | 2.09 (1.78,2.44) | 3.01 (2.56,3.51) | 2.63 (2.24,3.04) |

**Table A3.4:** Mean and bootstrapped 95% confidence intervals of contacts in work settings in each COVIMOD wave, stratified by *age risk* status.

|  | Mean contacts | |
| --- | --- | --- |
| Survey Wave | **Low risk:**  **Mean (95% CI)** | **High risk:**  **Mean (95% CI)** |
| POLYMOD | 11.32(10.39,12.23) | 1.42 (0.77,2.12) |
| 01: 30.04. – 06.05.2020 | 0.16 (0.12,0.22) | 0.1 (0.05,0.17) |
| 02: 14.05. – 21.05.2020 | 1.01 (0.73,1.37) | 0.76 (0.38,1.23) |
| 03: 28.05. – 04.06.2020 | 2.03 (1.32,2.74) | 1.25 (0.49,2.2) |
| 04: 11.06. – 22.06.2020 | 2.51 (1.93,3.18) | 1.13 (0.54,1.78) |
| 05: 26.06. – 01.07.2020 | 1.47 (1.07,1.94) | 0.98 (0.5,1.55) |
| 06: 09.07. – 16.07.2020 | 1.64 (1.12,2.2) | 0.62 (0.31,1.09) |
| 07: 24.07. – 29.07.2020 | 1.07 (0.64,1.6) | 0.38 (0.18,0.62) |
| 08: 07.08. – 11.08.2020 | 1.23 (0.72,1.95) | 0.52 (0.27,0.82) |
| 09: 04.09. – 09.09.2020 | 0.84 (0.52,1.25) | 0.19 (0.09,0.3) |
| 10: 30.09. – 05.10.2020 | 1.17 (0.61,1.89) | 0.24 (0.12,0.39) |
| 11: 14.10. – 21.10.2020 | 0.83 (0.61,1.09) | 0.55 (0.2,1.1) |
| 12: 29.10. – 03.11.2020 | 1.08 (0.66,1.55) | 0.87 (0.2,1.98) |
| 13: 05.11. – 10.11.2020 | 0.87 (0.57,1.23) | 0.2 (0.11,0.32) |
| 14: 25.11. – 30.11.2020 | 0.75 (0.52,1.01) | 0.27 (0.14,0.42) |
| 15: 09.12. – 15.12.2020 | 0.79 (0.48,1.23) | 0.14 (0.06,0.25) |
| 16: 23.12. – 30.12.2020 | 0.62 (0.31,1.03) | 0.11 (0.03,0.25) |
| 17: 28.01. – 02.02.2021 | 1.02 (0.43,1.8) | 0.28 (0.07,0.59) |
| 18: 24.02. – 03.03.2021 | 0.66 (0.37,1.04) | 0.4 (0.11,0.85) |
| 19: 17.03. – 26.03.2021 | 0.98 (0.48,1.62) | 0.25 (0.05,0.61) |
| 20: 07.04. – 15.04.2021 | 0.63 (0.34,0.99) | 0.37 (0.09,0.81) |
| 21: 12.05. – 24.05.2021 | 0.93 (0.71,1.16) | 0.31 (0.09,0.59) |
| 22: 26.05. – 03.06.2021 | 0.64 (0.47,0.85) | 0.24 (0.08,0.48) |
| 23: 09.06. – 22.06.2021 | 0.72 (0.5,0.98) | 0.39 (0.14,0.74) |
| 24: 07.07. – 19.07.2021 | 0.95 (0.65,1.32) | 0.38 (0.12,0.72) |
| 25: 04.08. – 13.08.2021 | 0.97 (0.72,1.27) | 0.27 (0.08,0.51) |
| 26: 01.09. – 14.09.2021 | 0.96 (0.72,1.27) | 0.27 (0.12,0.48) |
| 27: 22.09. – 06.10.2021 | 1.15 (0.9,1.41) | 0.54 (0.21,0.98) |
| 28: 08.10. – 20.10.2021 | 1.06 (0.83,1.33) | 0.47 (0.11,0.95) |
| 29: 22.10. – 02.11.2021 | 0.89 (0.69,1.12) | 0.32 (0.11,0.59) |
| 30: 03.11. – 09.11.2021 | 0.99 (0.74,1.27) | 0.3 (0.09,0.6) |
| 31: 17.11. – 23.11.2021 | 1.06 (0.77,1.41) | 0.18 (0.06,0.33) |
